# Bilateral, simultaneous, spontaneous rupture of patellar tendons without predisposing systemic disease or steroid use

**DOI:** 10.11604/pamj.2014.18.335.4218

**Published:** 2014-08-26

**Authors:** Rida-Allah Bassir, Aniss Chagou

**Affiliations:** 1 Mohamed V Souissi University, Department of Orthopaedic Surgery and Traumatology, Rabat, Morocco

**Keywords:** Patellar tendons, spontaneous rupture, systemic disease

## Image in medicine

Bilateral patellar tendon rupture is usually associated with systemic immunologic or connective tissue disease, steroid use, fluoroquinolone antibiotic use, or renal disease. Amongst the spontaneous cases, bilateral ruptures are exceedingly rare and have only been documented in a few case reports. We present a case of bilateral midsubstance patellar tendon ruptures along from a fall from a standing height in a 34-year-old otherwise healthy adult without any predisposing conditions, diagnosed in clinical examination with a little extension deficit, and confirmed in plain radiographs showing a bilateral patella alta (A, B). Most patients that sustain a tendon rupture have risk factors for tendinopathy including chronic renal disease, systemic lupus erythematosus, rheumatoid arthritis, or exposure to medications (such as fluoroquinolones or corticosteroids). To the best of our knowledge, this condition is often misdiagnosed. Despite the rarity of cases in patient's without systemic disease, emergency physicians must maintain a high index of suspicion of a knee extensor injury in a patient who is unable to actively extend the knees associated with patella alta.

**Figure 1:**
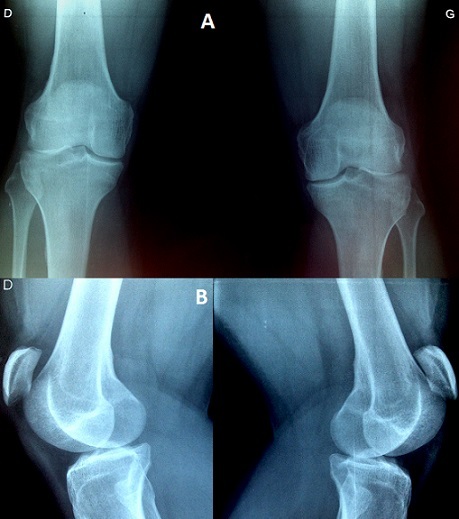
A) Bilateral frontal radiographs of the knee showing a patella alta; B) Bilateral lateral radiographs of the knee showing a patella alta

